# Machine learning to predict radiomics models of classical trigeminal neuralgia response to percutaneous balloon compression treatment

**DOI:** 10.3389/fneur.2024.1443124

**Published:** 2024-11-27

**Authors:** Ji Wu, Chengjian Qin, Yixuan Zhou, Xuanlei Wei, Deling Qin, Keyu Chen, Yuankun Cai, Lei Shen, Jingyi Yang, Dongyuan Xu, Songshan Chai, Nanxiang Xiong

**Affiliations:** ^1^Department of Neurosurgery, Zhongnan Hospital, Wuhan University, Wuhan, China; ^2^Department of Neurosurgery, Affiliated Hospital of Youjiang Medical University for Nationalities, Baise, China

**Keywords:** machine learning, nomogram, percutaneous balloon compression, trigeminal neuralgia, prognosis

## Abstract

**Background:**

Classic trigeminal neuralgia (CTN) seriously affects patients’ quality of life. Percutaneous balloon compression (PBC) is a surgical program for treating trigeminal neuralgia. But some patients are ineffective or relapse after treatment. The aim is to use machine learning to construct clinical imaging models to predict relapse after treatment (PBC).

**Methods:**

The clinical data and intraoperative balloon imaging data of CTN from January 2017 to August 2023 were retrospectively analyzed. The relationship between least absolute shrinkage and selection operator and random forest prediction of PBC postoperative recurrence, ROC curve and decision -decision curve analysis is used to evaluate the impact of imaging histology on TN recurrence.

**Results:**

Imaging features, like original_shape_Maximum2D, DiameterRow, Original_Shape_Elongation, etc. predict the prognosis of TN on PBC. The areas under roc curve were 0.812 and 0.874, respectively. The area under the ROC curve of the final model is 0.872. DCA and calibration curves show that nomogram has a promising future in clinical application.

**Conclusion:**

The combination of machine learning and clinical imaging and clinical information has the good potential of predicting PBC in CTN treatment. The efficacy of CTN is suitable for clinical applications of CTN patients after PBC.

## Introduction

Classic trigeminal neuralgia (CTN) is a spontaneous pain sensation that occurs in the trigeminal nerve region, and the clinical manifestation is mainly paroxysmal electric shock-like or pinprick-like recurrent pain, CTN is a type of chronic pain that excludes secondary causes such as tumors, multiple sclerosis, or arteriovenous malformations affecting the trigeminal nerve. CTN is characterized by sudden, severe, electric shock-like pain or tingling in the distribution of one or more branches of the trigeminal nerve (1). The exact cause of CTN is not fully understood, but it is usually associated with vascular compression of the trigeminal nerve root. This compression leads to demyelination of the nerve fibers, resulting in abnormal electrical conduction and hypersensitivity. CTN usually affects the side of the face with pain and may be triggered by daily activities such as chewing, talking, or touching the face. CTN is relatively rare, with an incidence of about 4–12 per 100,000 people per year. It is more common in women and is usually seen in individuals over the age of 50 (2, 3).

Surgical treatments for CTN include microvascular decompression (MVD), PBC, radiofrequency thermocoagulation and gamma knife radiation therapy. Each approach carries its unique risk–benefit profile, with treatment selection influenced by factors such as the patient’s financial situation, age, comorbidities, as well as patient and physician preferences (3, 4). At present, the anticonvulsants carbamazepine and oxcarbamazepine are the main drugs to treat TN, but medication can bring side effects including drowsiness, dizziness, skin rash, etc. Not only that, often patients will not be able to achieve the therapeutic dose of the medication or medication dosage should be considered for surgical treatment (1, 3). MVD is presently the preferred surgical intervention for trigeminal neuralgia when there is evident vascular nerve compression. However, it poses significant challenges to the surgeon and is associated with potential postoperative complications. These complications include exacerbated pain, cerebrospinal fluid leakage, intracranial infections, and other severe adverse events, which can ultimately result in patient mortality (4, 5). Among them, PBC is effective in relieving pain in many CTN patients. Studies have reported initial pain relief rates of 80–90%. The procedure is particularly valued for its rapid pain relief and relatively low risk of serious complications, also to treat patients with recurrence (6, 7). Therefore, clinicians are increasingly using PBC for patients who cannot tolerate other surgical methods (5). However, pain recurrence is common, and different recurrence rates are reported in the literature. Some patients may require repeat surgery to maintain pain relief. And common side effects of PBC include facial numbness, which may be temporary or permanent. Among the less common complications, patients may also experience bite muscle weakness, corneal numbness leading to keratitis, or numbness from anesthesia (painful numbness), infection, bleeding, or damage to surrounding structures (8). Therefore, Each approach carries its unique risk–benefit profile, with treatment selection influenced by factors such as the patient’s financial situation, age, comorbidities, as well as patient and physician preferences. Therefore, there is a need to develop a predictive tool to assess which patients are likely to achieve long-term remission from PBC before surgery, which will help in planning follow-up care and other interventions as necessary. This proactive approach could improve the sustainability of treatment benefits.

Radiomics is the use of data representation algorithms to extract and analyze image structural features from medical images provide detailed quantification of phenotypic and tissue heterogeneity, including tumors, allowing for a more precise and comprehensive assessment of disease (9–11). Machine Learning (ML) has revolutionized medical imaging by improving the ability to predict treatment outcomes for a wide range of diseases. By analyzing complex patterns in imaging data, ML algorithms can provide insights that inform personalized treatment plans and improve prognostic accuracy (12). PBC is a surgical procedure in which a needle is viewed through the cheek into the foramen ovale to compress the trigeminal ganglion using an intraoperative lateral fluoroscopic film. During the operation, the side perspective sheet shows the position of the balloon and the correct pear -shaped shape is considered the key to the success of the operation (13). Previous studies have shown that factors such as TN therapeutic effects and imaging features, and balloon shapes, volume and compression time may be related (14). Despite many scholars’ attempts to define the shape of the pear, the criteria for becoming a pear shape are still not uniform in the current position. Studies have reported the correlation analysis between clinical factors and the therapeutic efficacy of trigeminal neuralgia treatment (15). Most previous studies mainly focused on the comparative study of clinical data, ignoring the predictive value of imaging histology as well as histomorphology in disease progression. The predictive value of intraoperative balloon morphology imaging histological features for postoperative recurrence of TN is unclear.

In this study, patient clinical information and imaging histology morphological features based on intraoperative balloon x-ray were extracted, a predictive model for postoperative recurrence of TN was constructed by multifactorial logistic regression analysis, RF algorithms, a nomogram based on the columns of clinical risk factors and imaging histology features was established (16, 17). In conclusion, patients with CTN have considerable variability in their response to treatments such as percutaneous balloon compression (PBC). Our personalized prediction in this study could help identify which patients are most likely to benefit from PBC, thereby improving the overall success of the intervention.

## Methods

The study systematically collected clinical and intraoperative imaging data from patients with TN who underwent PBC between January 2017 and March 2023. Follow-up assessments were performed via telephone to monitor for TN recurrence. Inclusion criteria comprised patients aged 18 years or older diagnosed with primary TN, with complete clinical and imaging materials. The exclusion standards include the history of the surgical medical history of the previous microvascular decompression (MVD) or the glycerol root cutting (PGR) surgery (PGR) surgery, incomplete clinical or imaging records, secondary TN, recurrence TN, and patients who cannot be followed up after discharge (18).

In addition, pertinent clinical information, including collected clinical data, was obtained from the patient’s hospital medical record department. This information encompasses a comprehensive array of clinical data, including: age, gender, duration of facial pain, location of the affected side of the pain, trigeminal neuralgia typology, NRS score, evaluation of the efficacy of carbamazepine, balloon morphology, trigeminal nerve cardiac reflexes, facial numbness, weakness in mastication, keratitis, and score of the severity of compression. Clinical manifestations such as postoperative surgical response were confirmed and recorded by a Hao Mei imaging surgeon. Finally 117 patients were included in the study.

### Surgical procedure

All the procedures are consistent with what our team has done before (18). And analysis of the degree of vascular compression is performed post-procedure.

### Compression severity score

We define level I as mild blood vessels, and II and III levels are defined as severe blood vessels. Level I indicates that there are no blood vessels around the nerve or there are blood vessels around the nerve but there is no direct contact. Grade II indicates that the trigeminal is oppressed by blood vessels, but not to the extent that it causes distortion. Grade III is when a blood vessel compresses the trigeminal nerve, causing distortion. In the criteria of (18, 19).

### TN staging

Diagnosis was based on the International Classification of Headache Disorders (19). The TN classification is based on the standards formulated by the International Pain Research Association.

### Outcome assessment

Through the standardized telephone follow-up after the patient was discharged, the pain relief level was evaluated using the Barrow Institute of Neurology (BNI) Standard (I-V) (20, 21). We classify the BNI to II-III, indicating that the pain relieves it, and the BNI is graded to IV-V, indicating that the pain relief is invalid.

### Image segmentation and feature extraction

Intraoperative cranial X-ray images of randomly selected patients were evaluated for inter-observer agreement for feature extraction. Haematoma contouring was done manually by 2 experienced radiologists (film readers 1 and 2) independently without knowledge of clinical data. The reproducibility of inter-observer outlining of the region of interest (ROI) was evaluated using the intragroup correlation coefficient (ICC). ICC value>0.75 is considered to be a good and consistent instruction, using 3D Slicer software (Version 4.13) to extract the historical characteristics of all images. Image pre-processing, including noise removal, contrast enhancement and image smoothing, to improve image quality. Use thresholding, morphological operations, original_shape_maximum2DiameterRow: This is the length of the balloon in the direction of the maximum diameter. This can be determined by calculating the maximum distance between all pairs of points inside the balloon. original_shape_Elongation: This is the aspect ratio of the balloon and is used to describe how flat the balloon is. It can be determined by calculating the length of the long and short axes of the balloon (22).

### Imaging histological feature screening and model construction

One-way analyses were performed using R software, and clinical traits and imaging histologic features that were statistically different in a single factor were included in random forest (RF) tree model Screening important features and LASSO regression analysis for removing overfitting. RF parameter settings and validation. n_estimators: number of trees, usually 1000. max_depth: tree depth to avoid overfitting. 5 samples needed for re-splitting. Minimum number of samples for leaf nodes, increasing reduces overfitting. max_features: number of features considered per split, use sqrt(n_features). Cross-validation: k-fold cross-validation (*k* = 5) is used to assess the model’s generalization ability. The importance of each feature is assessed using Random Forest and the most important features are selected. Model performance was assessed using metrics such as *R*^2^ and MSE (23). LASSO regression: alpha is the penalty parameter, usually chosen through cross-validation. Normalize the data to ensure features are comparable. Cross-validation is used to select the optimal alpha value and to assess model performance. Examine residual plots to assess model fit (24). Calibration curves and DCA were used to assess the credibility of the model, and ROC curves (area under the ROC curve is greater than 0.5 and the diagnostic test has some diagnostic value) were used to validate the diagnostic ability of the model.

### Statistical analysis

Use R software for statistical analysis and data processing. Count data were expressed as percentages, while measurement data were presented as mean ± standard deviation. Group comparisons were conducted using t-tests or chi-square tests.

## Results

**Figure 1:**
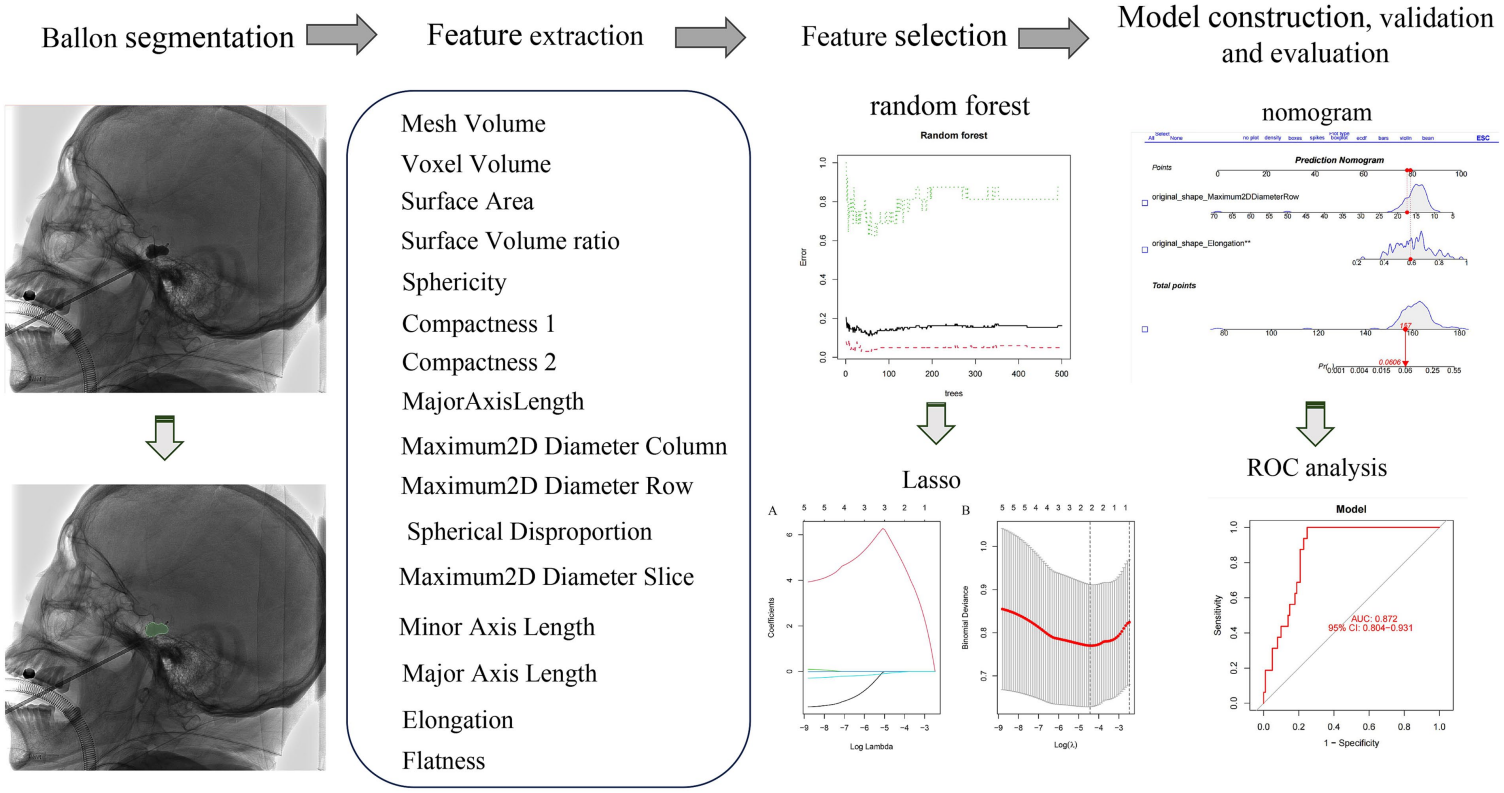
The overall workflow of our study. The ROI was manually drawn on the lateral plain film. Radiomic features were extracted from the radiographs to quantify their shape characteristics. LASSO and random forest algorithm were used to select radiomic features. A nomogram was created for individualized assessment, followed by ROC curve and decision curve analysis.

**Figure 2:**
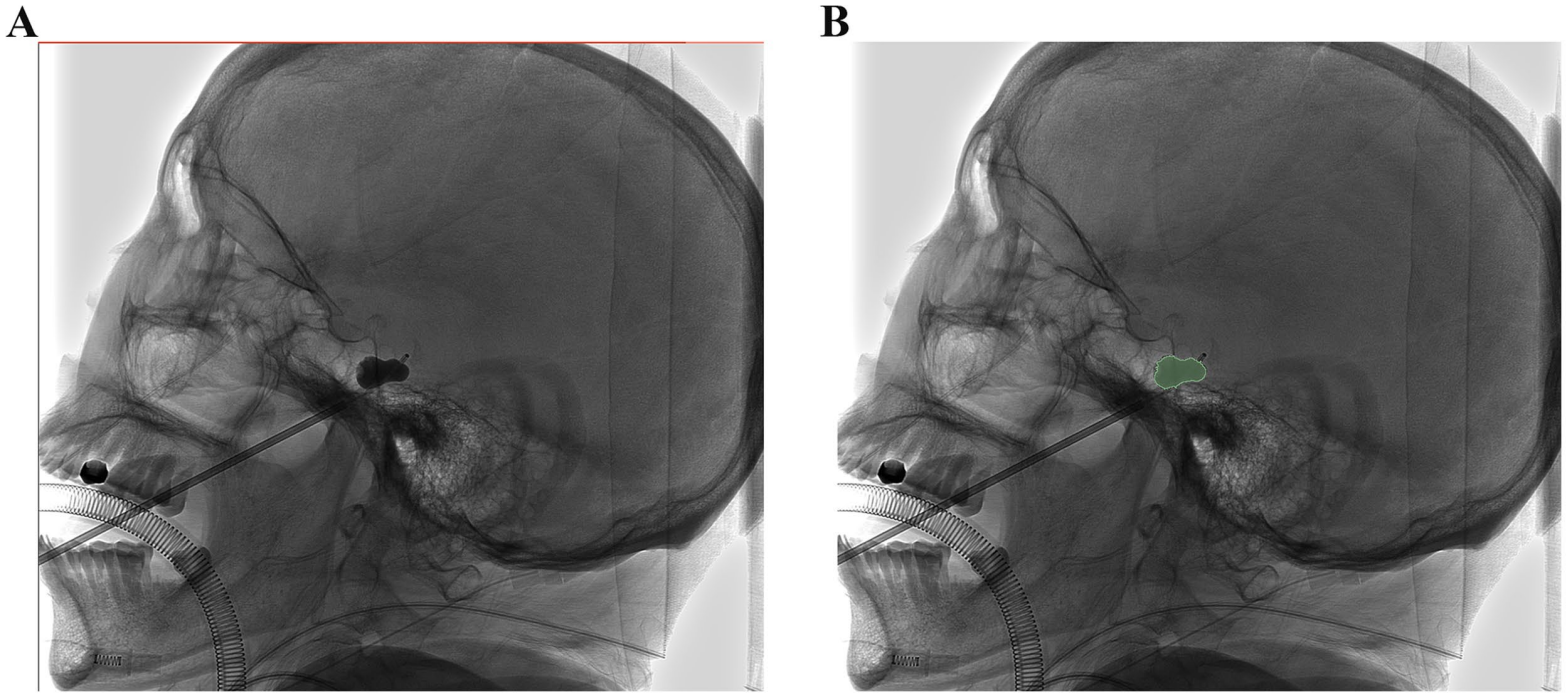
Image morphological feature extraction: **(A,B)** The ROI was manually drawn on the lateral plain film. Radiomic features were extracted from the radiographs to quantify their shape characteristics.

The study is depicted in Figure 1 as illustrated in Figure 2. LASSO and random forest algorithms were employed to select the most relevant radiomic features. Build nomogram for individual assessment, and then analyze the ROC curve and decision curve analysis to verify the reliability of the model.

### Clinical characteristics

A total of 117 patients diagnosed with Classical Trigeminal Neuralgia were included in the study, Among them, 43 men, 74 women, an average age of 69.11 (age is 48 to 95 years old). Table 1 summarizes the clinical characteristics of patients.

**Table 1:** Patient characteristics.

Characteristics	Ineffective group (*n* = 16)	Effective group (*n* = 101)	p
Age (year)	65.69 ± 10.849	69.69 ± 9.536	0.14
Symptom duration (months)	91.87 + 136.504	82.64 ± 116.866	0.70
Gender			0.937
Male	6 (37.5%)	37 (37.7%)	
Female	10 (62.5%)	64 (62.3%)	
Affected side			0.055
Right	13 (81.25%)	55 (77%)	
Left	3 (18.75%)	46 (23%)	
Trigeminal division			0.024
V1	0 (0%)	4 (3.96%)	
V2	7 (43.75%)	11 (10.89%)	
V3	1 (6.25%)	13 (12.87%)	
V1 + V2	5 (31.25%)	21 (20.79%)	
V1 + V3	0 (0%)	1 (0.99%)	
V2 + V3	1 (6.25%)	33 (32.67%)	
V1 + V2 + V3	2 (12.5%)	18 (17.82%)	
Compression severity score			0.50
No vessel near nerve or grade I	3 (18.75%)	20 (19.8%)	
Grade II or III	13 (81.25%)	81 (80.2%)	
Therapeutic effect of drugs			0.049
No response to medical treatment	12 (75.0%)	48 (47.52%)	
Multiple side effects on drugs	4 (25.0%)	53 (52.48%)	
Intraoperative trigeminal cardiac reflex			0.266
No	3 (18.75%)	68 (67.33%)	
Yes	13 (81.25%)	33 (32.67%)	
Postoperative complications			
Facial numbness			0.20
No	6 (37.5%)	57 (56.44%)	
Yes	10 (62.5%)	44 (43.56%)	
Keratitis			0.40
No	13 (81.25%)	90 (89.11%)	
Yes	3 (18.75%)	11 (10.89%)	

Among these patients, 16 cases were categorized into the ineffective group in the training set, while 101 cases were classified into the effective group. There are no statistical differences in the two groups of patients in the age, duration of symptoms, accumulated sides, digital evaluation meters (NRS), trigeminal nerve reflexes, facial numbness, and keratitis (Table 1).

### Univariate analysis for TN outcome

The relationship between the clinical characteristics of one -way logic regression and the prognosis of TN is adopted. As shown in Table 2, we noticed a significant correlation between the treatment effect between the trigeminal nerve division and the training concentrated drug TN ending (*p* < 0.05). Females accounted for the majority of patients who relapsed, and TTN strategy division is an important prediction indicator immediately relieved (or 0.70, 95%CI 0.51 ~ 0.94; or 0.71, 95%ci 0.51 ~ 0.95).

**Table 2:** Univariate and multivariate logistic analyses of prognostic risk factors for TN.

Variable	Univariate logistic analysis				Multivariate logistic analysis			
	OR	(95% CI)		*p* value	OR	(95% CI)		*p* value
Sex	1.04	0.33	3.03	0.9				
Age	0.96	0.91	1.01	0.14				
Affected side	0.28	0.06	0.92	0.055				
Trigeminal division	0.70	0.51	0.94	0.024	0.71	0.51	0.95	0.028
Compression severity score	0.78	0.36	1.68	0.5				
Intraoperative trigeminal cardiac reflex	2.10	0.63	9.63	0.3				
Facial numbness	2.16	0.74	6.77	0.2				
Therapeutic effect of drugs	0.30	0.08	0.93	0.049	0.31	0.08	0.97	0.058
Keratitis	1.89	0.39	7.06	0.4				

### Machine learning to build diagnostic models and validation

**Figure 3:**
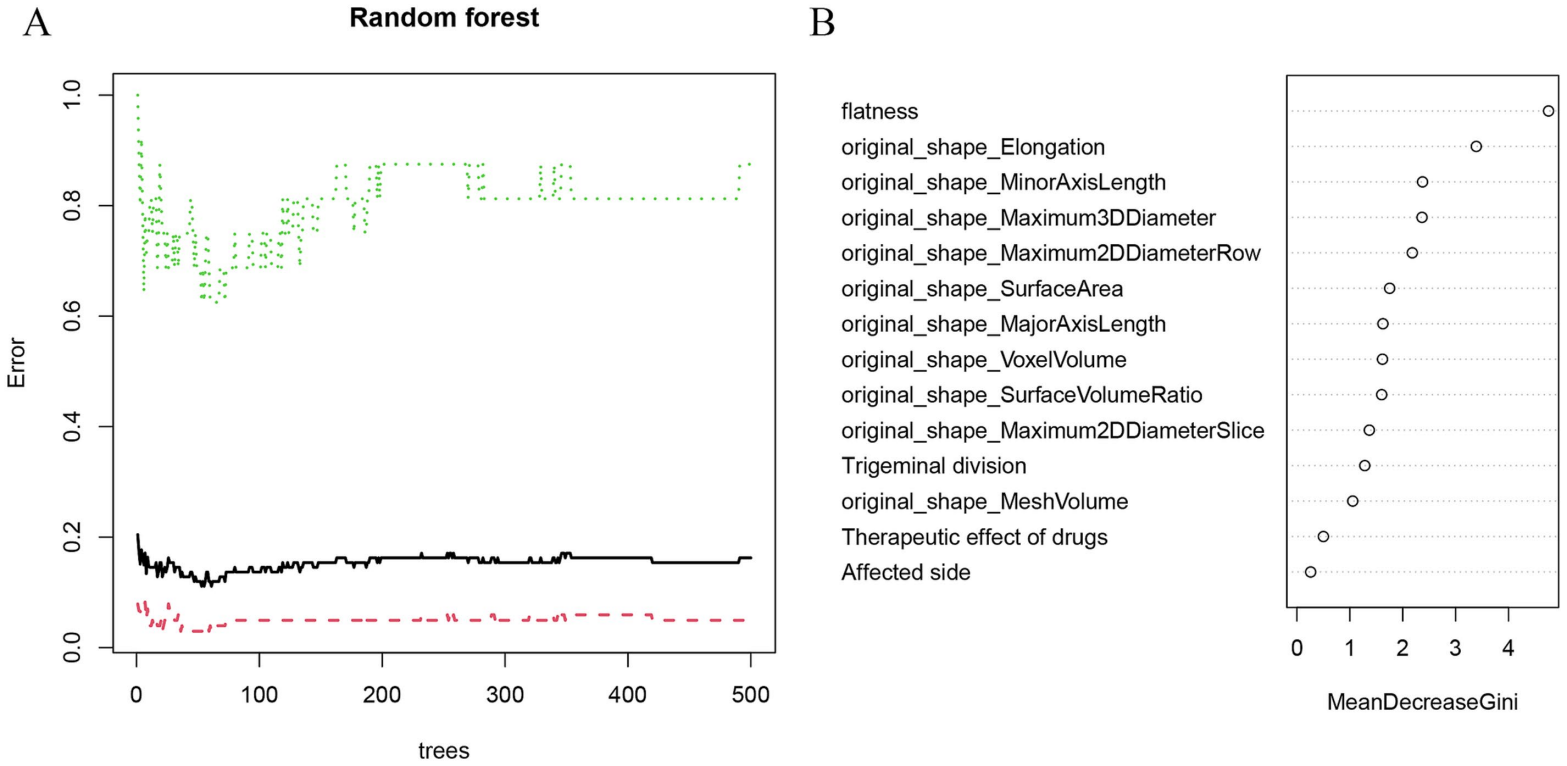
Random forest screening to predict the characteristic factors of recurrence. **(A)** The random forest algorithm showed the error between the relapse group and the control group. **(B)** Rank clinical-imaging morphological features according to importance scores.

**Figure 4:**
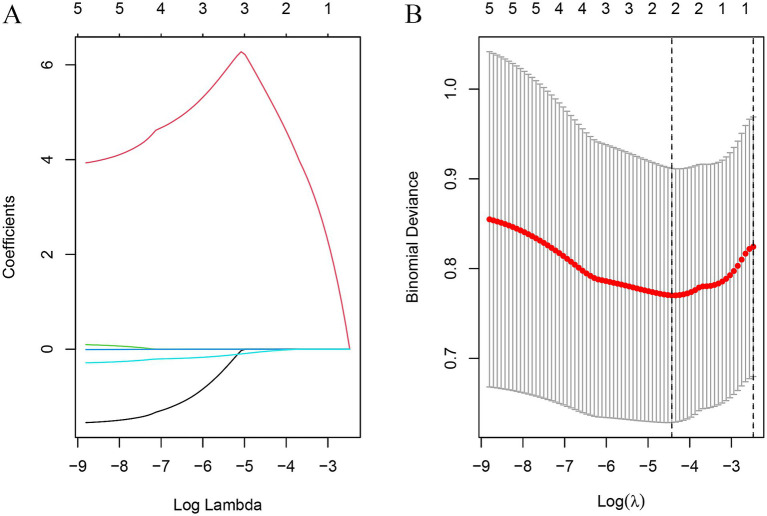
Texture feature selection using the least absolute shrinkage and selection operator (LASSO) logistic regression **(A)** The optimal tuning parameter (*λ*) was selected using 10-fold cross-validation in the LASSO regression model; **(B)** LASSO regression coefficient distribution.

**Figure 5:**
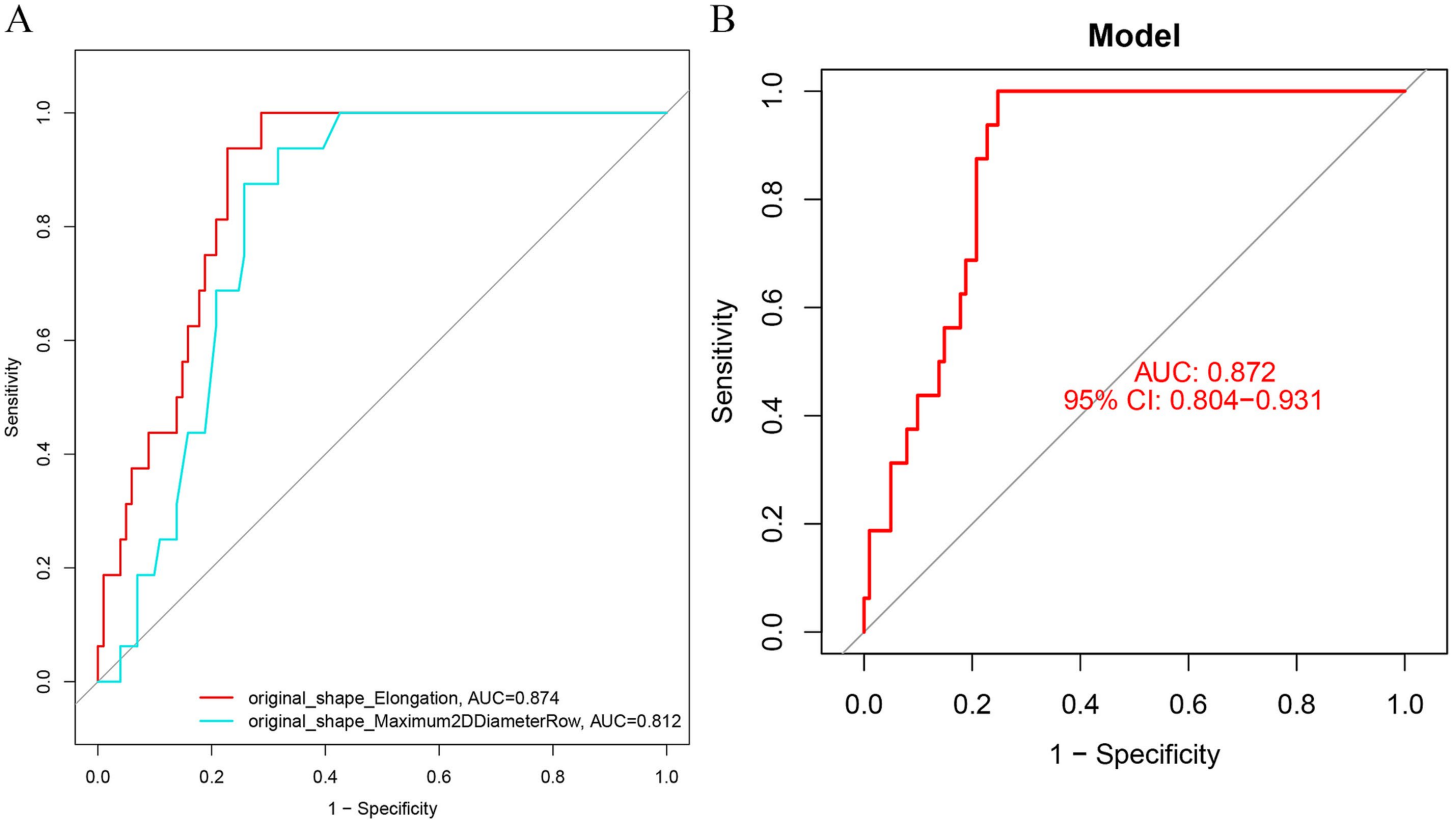
**(A)** ROC curve was used to analyze the prediction efficiency of image morphological feature model **(B)** ROC analysis of diagnostic model in training set. **(B)** ROC analysis of diagnostic model in validation set. AUC, area under the curve; subject operating characteristic curve (ROC); CI. Confidence Interval; LASSO, Least Absolute Shrinkage and Selection Operation.

Initially, three radiomics features were differentially extracted from the depicted ROIs. The inter-observer ICC ranged from 0.751 to 0.997, indicating good reproducibility of these features. Subsequently, the best radiomics features were identified from the ROI through mRMR and random forest (RF) tree model screening important features, as illustrated in Figures 3A,B. We performed a lasso Add a penalty function to keep compressing the coefficients so as to streamline the model to avoid covariance and overfitting (Figures 4A,B). The ROC curves of each feature in the model are shown, and their AUC values are:

original_shape_Maximum2D DiameterRow, original_shape_Elongation were screened to predict the prognostic prediction of TN for PBC treatment with the areas under the roc curves being. 0.812, 0.874; and the predictive and under the ROC curve of the model is 0.872 (Figures 5A,B).

## 4. Establishment of radiomics model

**Figure 6:**
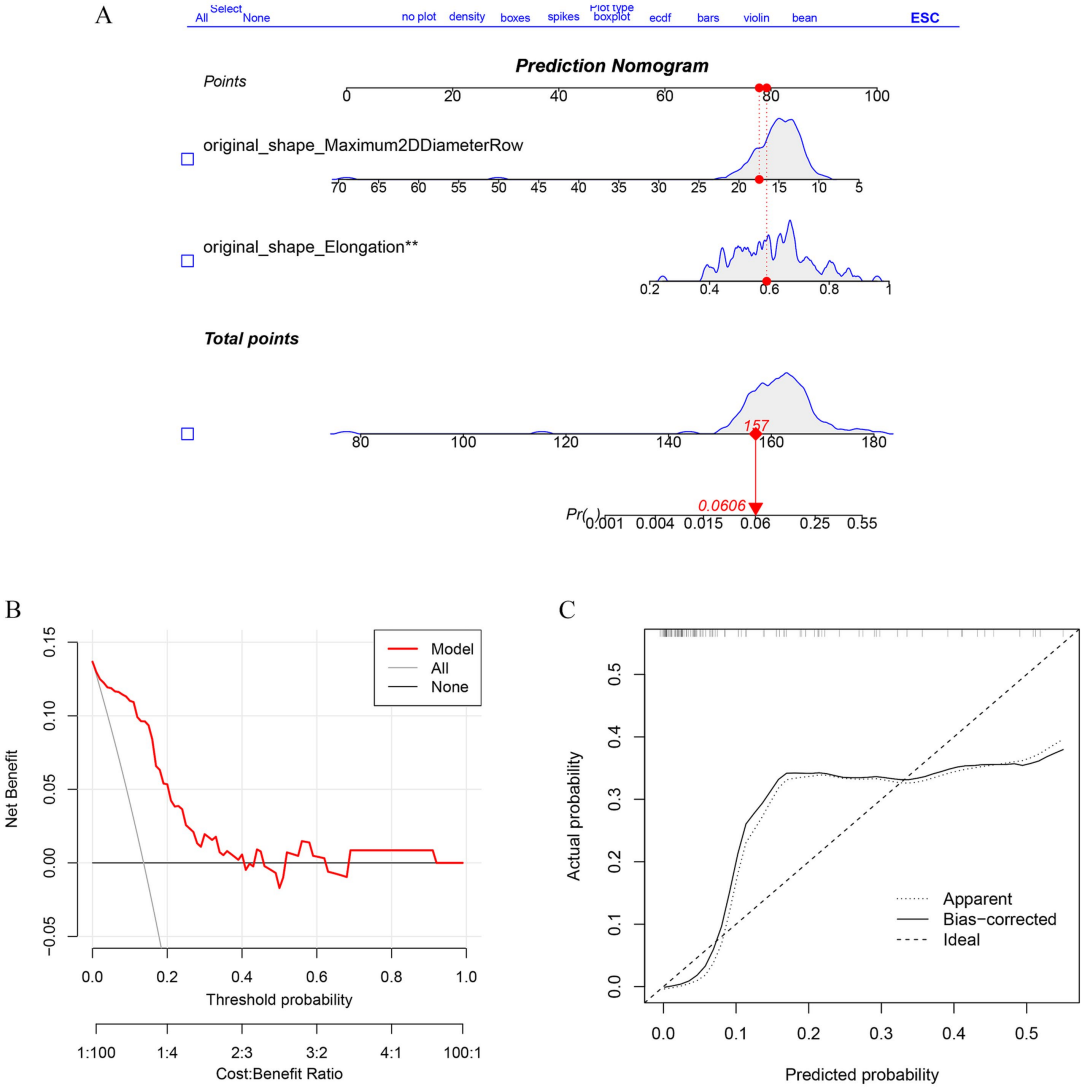
Establishment of Radiomics Model and performance verification. **(A)** Nomogram was constructed to predict the probability in recurrent trigeminal neuralgia progress. The values of each variable (Affected side, sex, Flatness, Major Axis Length) are summed to obtain a total score. **(B)** The calibration curves of the nomogram. **(C)** The DCA curve shows the clinical decision benefit of this model.

Imaging features from the RF model and lasso:original_shape_Maximum2DDiameterRow,original_shape_Elongation were incorporated to predict the risk of TN recurrence. A nomogram predicting the probability of effective regression after PBC in a typical TN patient was established (Figure 6A), and the calibration curve also shows the consistency of satisfactory (Figure 6B). The decision -making curve shows that the pillar diagram of the risk of effective results increases more benefits than the full or no scene (Figure 6C).

## Discussion

Classical trigeminal neuralgia (TN) is characterized by sudden, severe, electric shock-like or stabbing pain in specific areas of the face, typically on one side and affecting the trigeminal nerve’s V2 and V3 branches. These brief episodes, lasting seconds to minutes, can occur multiple times a day and are often triggered by routine activities like chewing, speaking, or touching the face (1, 4). However, due to the chronic onset of pain, when the pain is severe, it will seriously affect the patient’s normal life and work (2). In recent years, PBC has become a common therapeutic option for the treatment of TN, which has a high success rate, simple technique, and relatively low risk, especially in elderly patients (25). PBC relieves trigeminal nerve pain by damaging myelinated axons involved in pain transmission, causing focal axonal injury and blocking abnormal discharge pathways (26). However, PBC has several disadvantages. Potential complications include facial numbness, which can be permanent, and sensory deficits. The procedure may also result in temporary or permanent jaw muscle weakness. Additionally, there is a risk of infection, bleeding, or hematoma at the puncture site. Over time, axonal myelin sheaths may regenerate, leading to pain recurrence (27). Previous studies indicate that PBC provides immediate postoperative pain relief in 82–97.1% of cases, lasting about 18–20 months (5, 28, 29).

Percutaneous balloon compression (PBC) stands as a minimally invasive procedure frequently employed to address trigeminal neuralgia (TN). Its mechanism involves compressing the trigeminal ganglion, thereby mitigating pain by impairing the nerve fibers responsible for pain signal transmission. Despite its effectiveness, PBC can lead to various complications. Among these, facial numbness emerges as the most prevalent. While indicative of successful trigeminal ganglion compression, persistent or severe facial numbness can significantly impact quality of life (13, 29, 30). Several previous studies have shown that clinical factors such as preoperative TN type, symptom duration, NRS, and TN nerve compression severity scores in patients with atypical pain affect the outcome of ablative procedures including GR and SRS, as well as the consequences of MVD procedures (13, 30, 31). The treatment of PBC in CTN patients is still largely dependent on subjective judgment (32, 33), and therefore there is a need to develop a validated method for predicting recurrence modeling after PBC. Leveraging radiomics could offer a non-invasive avenue to discern which patients are most likely to benefit from PBC by analyzing pre-procedure imaging data. Such an approach could pave the way for personalized treatment plans and improved outcomes in CTN patients.

Radiomics has substantial advantages over traditional structural imaging by artificial intelligence applications to develop predictive models (34–36). This integration significantly improves clinicians’ capacity to make evidence-based diagnoses and predict treatment efficacy. The response to treatments like PBC varies widely among patients with Classical Trigeminal Neuralgia (CTN). Individualized predictions can aid in identifying which patients are most likely to benefit from PBC, thereby improving the overall success of the intervention. By predicting treatment response, clinicians can select appropriate candidates for PBC, potentially avoiding invasive procedures for those unlikely to benefit (29, 37). In our study, we identified two crucial imaging features and developed an accurate prediction model utilizing LASSO and random forest algorithms. This model was specifically designed to predict the outcome of postoperative PBC in patients with CTN, achieving an AUC of 0.872, indicative of good performance. Furthermore, the calibration curve exhibited good agreement with the decision curve analysis (DCA) curve, suggesting potential clinical application. This imaging histology model could transform patient management. The model identifies high-risk patients by combining imaging and histological features with clinical factors. This risk assessment helps clinicians develop personalized treatment plans, offering early intervention or alternative options for those with a poorer prognosis. Treatment decisions can be made by identifying patients who are more likely to benefit, thereby optimizing the allocation of resources (38).

The clinical imaging histological model developed in this study predicted the efficacy of PBC in patients with trigeminal neuralgia (TN), but has limitations. The sample size was small and the data were derived from a single center, limiting the generalisability and applicability of the findings. The selection of imaging histological features may be limited by the accuracy and consistency of the imaging technology used. Future studies should focus on multi-center studies to validate the model. Combining clinical, genomic and other biomarker data improves the accuracy and reliability of predictive models. Examining patients’ clinical and imaging characteristics over time allows for more precise and individualized treatment. By addressing these limitations, imaging genomics models can be enhanced, ultimately improving patient prognosis and outcomes.

## Conclusion

The use of radiological features and machine learning models to predict treatment response in CTN is essential for personalized treatment planning. This approach is expected to optimize treatment outcomes and reduce adverse effects, as well as improve patient quality of life and cost-effectiveness. The subsequent construction of large-sample multicentre predictive modeling is expected to transform CTN management into a more precise and patient-centered practice, ultimately leading to better clinical outcomes and higher patient satisfaction.

## Data Availability

The original contributions presented in the study are included in the article/supplementary material, further inquiries can be directed to the corresponding authors.
